# Digital Electronic System-on-Chip Design: Methodologies, Tools, Evolution, and Trends

**DOI:** 10.3390/mi15020247

**Published:** 2024-02-07

**Authors:** Marcian Cirstea, Khaled Benkrid, Andrei Dinu, Romeo Ghiriti, Dorin Petreus

**Affiliations:** 1 School of Computing and Information Science, Anglia Ruskin University, East Road, Cambridge CB1 1PT, UK; 2 Arm Ltd., 110 Fulbourn Rd, Cambridge CB1 9NJ, UK; 3 Collins Aerospace, Fore 3, Huskisson Way, Stratford Road, Shirley B90 4SS, UK; 4 Exquisite IT Ltd., 33 Stokes Drive, Huntingdon PE29 2UW, UK; 5 Applied Electronics Department, Technical University of Cluj-Napoca, Baritiu Street, 400027 Cluj-Napoca, Romania; dorin.petreus@ael.utcluj.ro

**Keywords:** system-on-chip (SoC), design methodology, field programmable gate array (FPGA), electronic design automation (EDA), electronic system level (ESL) design, high level synthesis (HLS), artificial intelligence (AI), machine learning (ML), generative design, prompt engineering

## Abstract

This paper reviews the evolution of methodologies and tools for modeling, simulation, and design of digital electronic system-on-chip (SoC) implementations, with a focus on industrial electronics applications. Key technological, economic, and geopolitical trends are presented at the outset, before reviewing SoC design methodologies and tools. The fundamentals of SoC design flows are laid out. The paper then exposes the crucial role of the intellectual property (IP) industry in the relentless improvements in performance, power, area, and cost (PPAC) attributes of SoCs. High abstraction levels in design capture and increasingly automated design tools (e.g., for verification and validation, synthesis, place, and route) continue to push the boundaries. Aerospace and automotive domains are included as brief case studies. This paper also presents current and future trends in SoC design and implementation including the rising, evolution, and usage of machine learning (ML) and artificial intelligence (AI) algorithms, techniques, and tools, which promise even greater PPAC optimizations.

## 1. Introduction

The relentless march of Moore’s law [1] has enabled the computing industry to provide exponential increases in computing power to consumers globally, throughout the last six decades. The underlying developments in semiconductor technologies (such as in materials, laser optics, lithography) are now enabling atomic-size miniaturization levels with billions of transistors integrated into a single chip. The design methodologies of these chips have also had to evolve markedly over several decades. From manual design in the early years of the industry, to computer-aided design (CAD), and now to artificial intelligence (AI)-assisted electronic design automation (EDA) tools, none of the above progress could have happened without this evolution in design methodologies. These days, EDA tools are supporting the design of multi-die system-on-chips (SoCs), optimizing for billions of parameters to meet specific performance, power, area, cost (PPAC) constraints.

In order to achieve an enabling design environment that allows simultaneous consideration of all engineering system parameters at an early stage in the design process, holistic modelling and simulation of complex electronic systems is key. The use of high-level languages enables the evaluation of an idea in the early stages of the design process, as they allow the initiation/setup of a test environment at an abstract functional level. Therefore, they constitute an appropriate solution for the description of novel designs. The outcome of this system-level approach is a design environment based on modern EDA tools that enables an early-stage hardware/software partitioning of the design along with functional simulation ahead of synthesis, timing analysis, and verification, hence optimizing for specific PPAC sweet spots. We summarize the evolution of electronic systems over the decades along the following parameters (DIGIT):

○ **D**own-up or bottom-up: refers to the traditional way in which electronic systems were designed and built, i.e., starting at the bottom of an imaginary pyramid of building blocks and leading up to a complete system.
○ **I**nformatics: the explosion of data and information brought about by the evolution of memory and communications technologies, including the Internet, has led to a rising demand for increasingly complex electronic systems.
○ **G**lobalization: the relentless march of globalization since the 1980s has led to extreme specialization in various parts of supply chains, which in turn led to huge productivity gains leading to further pressures on complexity of use cases and underlying new electronic systems.
○ **I**ntegration: the above led to the integration of more functionality into electronic systems, including chips which became system-on-chips over time, with various types of processors to suit multiple needs, e.g., CPUs, GPUs, DSPs; analogue front ends, e.g., for sensors or radio receivers; various memory types; transceivers; and switch fabrics. These are now increasingly built from chiplets integrated together in 2.5D or 3D under a single package.
○ **T**op-down: the above is driving more top-down design methodologies, making use of higher levels of abstraction in design specification, optimization, verification, validation, and testing.

The remainder of this paper will revise the evolution of EDA methodologies, tools, and target technologies, outline the fundamentals of SoC design flows in industrial electronics, and showcase the crucial role of the intellectual property (IP) industry. Two application domains were chosen as case studies to briefly demonstrate the above evolution, aerospace, and automotive engineering, before outlining emerging/future trends in electronic design automation. A summary will finally be provided.

## 2. Overview of System-on-Chip Design Methodologies and Tools

### 2.1. Brief Historical Evolution of Electronic Systems Design Methodologies and Tools

As mentioned earlier, the last six decades saw an unprecedented evolution of computational capacity, both in terms of software and hardware, with an exponential growth of the integration scale of silicon devices targeted for electronic circuit hardware implementation (following Moore’s Law). Another major advent was the apparition and exponential growth of the Internet. To cope with this growth, we have also seen an evolution of computer-aided engineering design tools for electronics, now known as electronic design automation (EDA) tools, which have evolved from ‘islands of automation’ to integrated and automated design packages, able to handle higher levels of abstraction and complexity [2].

A range of early initiatives/developments aimed to achieve an overall engineering systems modeling environment at higher levels of abstraction, enabling the creation of a test environment early in the design cycle. This allowed for more holistic evaluation of engineering systems with optimized integrated functionality, further enabling the more detailed design of its components. Just a few attempts are mentioned here. Rosetta was an IEEE initiative (1999) of system-level design language that allowed users to create reusable components that specify the structure and behaviors of their system and subsystems. It targeted the modeling of engineering systems that combined complex electronics with complex mechanical systems. The Millenium Machine (2000) was a pioneering initiative of the Engineering and Physical Sciences Research Council (EPSRC), UK, for holistic modeling of engineering systems (systems of systems). The aim was to achieve a fully digital model of an engineering system (for example a car or an aircraft). The more recent Modelica is an object-oriented equation-based modeling language for complex physical systems containing mechanical, electrical [3], electronic, hydraulic, thermal [4], control, power/energy [5], or process-oriented components. It allows, for example, the graphical modeling of electronic components (linked to SPICE) or mechanical parts (Dymola, offering models for CATIA).

Another step was the evolution of tools such as MATLAB and Simulink, used frequently in electronic systems’ mathematical modeling, which had dedicated toolboxes added, thus streamlining the hardware implementation of digital controllers associated with the system modeled. The latter could be electronic, electrical, and/or mechatronic, therefore bridging the gap between mathematical modeling and hardware prototyping tools, such as those based on microprocessor/DSP compilers or hardware description languages (HDLs) [6] and facilitating hardware implementation in field programmable gate arrays (FPGAs) or application-specific integrated circuits (ASICs). Due to the increased availability of FPGAs, which contain microprocessor/DSP cores, as multi-million-gate digital and reconfigurable system-on-chip, design methods had to adapt to enable faster time-to-market for complex products.

**Figure 1:**
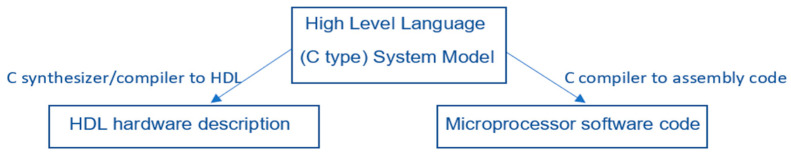
Principle of top-down hardware/software co-design.

Such examples, although not exhaustive, illustrate a trend towards EDA tools integration and their evolution towards a higher level of abstraction, in order to address top-down electronic systems’ complex functionality and achieve combined/integrated optimization [2]. Through such EDA tools, an electronic system’s functional description and its electronic design details are rigorously combined ahead of implementation. A top-down holistic design approach (Figure 1) enables engineering systems and associated electronic controllers to be developed faster and offers added benefits by creating a test environment at an early stage in the design cycle to evaluate system performance. They also generate reusable design modules and enable hardware/software co-design [7].

### 2.2. Electronic Systems—Design Methods and Target Technologies

Reflecting on the evolution of design methods, after the transition of methodologies of electronics systems from a bottom-up approach, based on schematic entry (netlist) of the intended circuit, to an HDL-based top-down approach, the next significant step change was marked by electronic system level (ESL) design methods. It can be said that these render an even higher level of abstraction, handle increased complexities at system level, and enable the implementation of digital systems combining hardware and software solutions. In the context of ESL design methods, this subsection also covers the emergence of FPGAs and ASICs as target implementation technologies.

#### 2.2.1. Text-Based High-Level Synthesis (HLS) Design Methods

A first category design methodologies is represented by those taking an ESL approach, by making use of text-based HLS tools [8], which enable the behavioral system description developed using a high-level language (such as System-C) to be transformed into a register transfer level (RTL) implementation/description [9,10]. In this approach, adopted in many industrial applications targeting FPGAs [11], the underlying details of implementation are not visible to the user and their automatic generation enables a shorter time to market and reduces the fabrication costs.

In the evolution of design methods and tools, the flexibility and choice of modeling styles offered by HDLs became insufficient at some stage. C-Synthesizer and System-C are examples of more efficient system design environments/compilers, which allow high-level language modeling/simulation/design of electronics/mechatronics systems and electronic controller system-on-chip implementation, with power efficiencies, especially relevant to Internet of Things (IoT) applications with microcontrollers and FPGAs often as popular targets [12,13]. Hardware/software co-design is also facilitated [14] in a holistic approach. With increased system complexity, network-on-chip (NoC) technology emerged as an effective and efficient system component [15,16].

#### 2.2.2. Schematic-Based/Graphical Electronic System Level (ESL) Design Methods

A second category of ESL design approaches is that based on schematic or visual programming. The tools facilitating the top-down approach enable the design to be developed at high level of abstraction, in a graphical format. There are two popular families of tools in this category, namely the Mathworks family, which includes Matlab [17,18,19] and Simulink [20,21], and the National Instruments (NI) family, which includes Labview [22,23,24] and can make use of third-party tools such as ModelSim [25,26]. They provide component libraries for the modeling and simulation of electronic and mechatronic systems and include models of semiconductors, motors, drives, sensors, and actuators [27].

From such tools, it is possible to deploy models to other simulation platforms. C-code or HDL generation is possible, too, thus facilitating direct hardware implementation of complex digital systems. In combination with FPGA technology for implementation, such tools also facilitate the hardware-in-the-loop (HIL) approach to electronic systems design [28,29], where part of the system being designed and developed (either the controller or the controlled plant) is modelled and simulated in conjunction with real hardware elements, thus enabling an effective evaluation and validation of a complex electronic/engineering system’s behavior. This makes use of the novel concept of ‘Digital Twin’ (DT) [30,31,32]—a virtual representation serving as real-time digital counterpart of a physical engineering system or functional process. DTs are essentially using real-time data from sensors to monitor a physical object. Their use can improve efficiency and predict future performance. DTs are nowadays increasingly used by organizations such as Ocado, Rolls-Royce, NASA, and Ford [32] or for managing risk in security critical environments [33].

#### 2.2.3. FPGAs and ASICs

In the 1980s, a new underlying computing technology emerged, FPGA, initially as interface with microprocessors (undertaking tasks that can render themselves to parallel processing). In its basic form, an FPGA is defined as a matrix of interconnected configurable logic blocks (CLBs) [34]. In most cases, these hardware structures are reprogrammable (can be reconfigured), several such technologies being available (Flash, EPROM, SRAM). Since then, a range of FPGA devices have been developed and they have grown to a technology of established maturity [30]. These have initially enabled rapid prototyping of products but nowadays are more and more used as mainstream implementation target. Recent FPGAs also feature A/D converters and fast I/Os, enabling easy integration with mixed analog/digital peripherals, and high-speed transceivers. Such devices are generally suited for high speed and demanding industry applications.

Some design tools associated with this technology are very user friendly; designers can develop hardware architectures dedicated to the implementation of a certain control algorithm. The hardware validation of the design, following the earlier stages of circuit design and simulation, is facilitated by the use of FPGA, either as permanent hardware target of the product or as prototyping ahead of a permanent chip implementation in an ASIC.

Thus, re-programmable FPGA devices provide a fast and relatively low-cost method to validate electronic controllers and system designs, in conjunction with modern EDA tools, leading quickly to well-verified designs ahead of final implementation. Following historical developments, nowadays complex FPGAs also include high end microprocessor cores [35], thus eliminating the dilemma of choosing between hardware fabric or a processor-based architecture; the ‘right’ hardware target device for a digital electronic controller is often an embedded system-on-chip [30].

### 2.3. SoC Design Methodologies and Tools—Users and Enablers of AI

The use of EDA tools and techniques for electronic systems design facilitates easy development and hardware implementation of intelligent controllers employing complex algorithms, leading to AI-based designs for industrial applications [36,37,38,39,40]. Their quick route to hardware and the flexibility offered, especially through embedded system-on-chips, enable hardware/software co-design and implementation [41].

The rise of deep neural networks (DNNs) driven by higher computational capabilities and improved algorithmics has allowed for more embedded and powerful intelligence in modern SoCs. Moreover, hardware-based neural networks are offering to industry higher performance and low power consumption compared to pure software implementations, exploiting the vast parallelization opportunities offered by DNN computations, and they can be embedded in a wide range of systems [38]. Cost is being addressed by the scale of consumer electronics harnessing this technology as well as the exponential growth in cloud computing. It is worth noting that FPGAs often occupy an interesting PPAC sweet spot.

High-level descriptions of the neural algorithms in an industry standard that allows full simulations and fabrication also constitute essential aspects for achieving advanced AI systems. Traditional machine learning (ML) as well as DNN models can be captured in tools such as MATLAB software libraries. Python or C-based high-level hardware languages constitute other popular formats [37,39].

## 3. Industrial SoC Design

### 3.1. Fundamentals of SoC Design Flows

**Figure 2:**
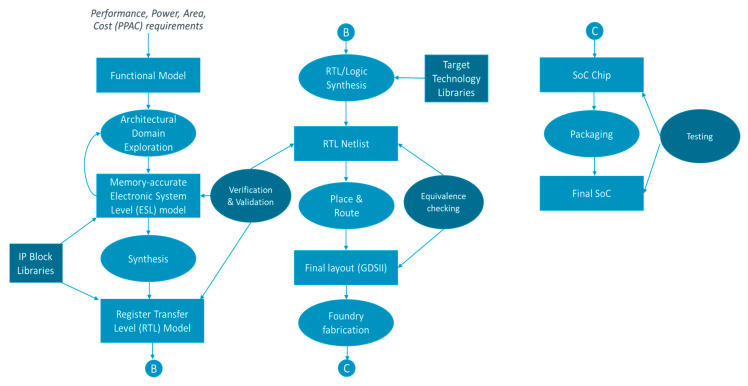
A high-level view of a SoC design flow.

As tool outline, Figure 2 gives a high-level view of a typical SoC design flow. In such flow, firstly, high level requirements are distilled into functionality with PPAC requirements of the desired SoC. Many inputs are needed for this to happen, including the following:

- Marketing requirements: a gap is found in market offerings, e.g., an unserved segment of user needs, or opportunity for differentiation on cost or functionality compared to competition.
- Incremental improvements from previous products, e.g., moving to next generation semiconductor node, or leveraging a new memory or interconnect technology.
- New application area requirements, e.g., a new machine learning (ML)/artificial intelligence (AI) paradigm which can benefit from custom acceleration.

The above are usually captured in high level language with some formal rules, document templates for example, or an engineering requirement management tool such as SysML [42]. A functional model is then developed to capture the required functionality, i.e., what the SoC should produce in response to various user inputs, for example. This is usually conducted using a high-level language (C/C++) with low-level implementation details (block architecture, execution speed, or power consumption) abstracted away. What follows is an iterative process of architectural domain exploration which partitions the systems into various blocks including processors, domain-specific co-processors or accelerators, memory, interconnects, and communication interfaces. Design space exploration depends on many inter-related technical and non-technical inputs, linked to questions like [43]:

- Which processor(s) or core(s) to use? Standard or custom, self-developed or from third party?
- Which co-processors, if any, and which peripherals to use? Standard or custom, self-developed or from third party? Such co-processors include graphic processing units (GPUs), digital signal processors (DSPs), and neural processing units (NPUs).
- Which software stack to use? This includes operating system choice if any, and availability of various software stack layers. This choice is intimately related to the choice of processors/co-processors.
- Which memory types and hierarchy to use? Standard or custom, self-developed or from third party?
- Which type of interconnect to use? Bus architecture or network-on-chip, standard or custom, self-developed or from third party?
- Security of supply including support, e.g., if a third-party supplier of a component fails to deliver, for any reason, is there at least a second source supplier? Geopolitical turmoil is playing an increasingly important role in the choice of suppliers these days as export controls make certain technologies and suppliers impossible to access. In some industries, such as automotive, security of supply and support over a long period of time is a fundamental requirement.

The reuse of existing intellectual property (IP) blocks and corresponding high-level models has been an important driver of higher performance and higher quality SoCs at economic prices. Reuse of various software stacks is also another important driver. Throughout the process, PPAC constraints are at the forefront of the system architect’s mind as they make partitioning decisions, initial estimates of performance, power, area, and cost consequences of particular choices, iterating before settling on a partition that meets or is as close as possible to the desired PPAC requirements.

Once a partition has been chosen, an electronic system level (ESL) model is produced. This is captured in languages such as SystemC [44] or SystemVerilog [45]. High level synthesis then takes over. This includes the following:

- Logic synthesis: Converting high level ESL models into register-transfer level (RTL) descriptions of logic e.g., processor, glue logic. Verilog [46] or VHDL [47] hardware description languages (HDLs) are generally used to capture RTL. The reuse of pre-designed pre-verified IP blocks is popular for faster time to market and economic efficiency. High-level synthesis (HLS) tools are also sometimes used to generate RTL from high level languages, e.g., C/C++ [48] or Python [49], although this is mostly performed in field programmable gate array (FPGA) designs [50]. Note that the RTL generated in this phase is called behavioral RTL, as it does not depend on any underlying implementation technology yet. The next synthesis phase will take the implementation technology into account.
- Memory synthesis: converting high level memory transaction models into memory blocks with input/output ports, bit width information etc.
- Interconnect synthesis: Interconnect plays a crucial role in meeting PPAC constraints; for example, logic occupancy can be severely impacted if logic cannot access the right data at the right time. This step is about converting the high-level communication needs into the right interconnect architecture, e.g., bus hierarchy or network-on-chip (NoC), with commensurate details. Here again, reuse of pre-designed pre-verified interconnect is popular.

The next phase is to synthesize RTL with target technology information. These are usually available in the form of technology libraries, e.g., basic logic or memory block/cells. This phase also includes clock and power/ground network synthesis. Tools used for this phase include Synopsys Design Compiler [51] or Cadence Genus RTL Synthesis [52]. The result is a technology-informed RTL netlist.

Throughout the above process, validation and verification steps using simulation models and sometimes formal methods take place to make sure every iteration model is consistent with previous stage models and with the SoC functional requirements. Testbenches with constraints derived from functional requirements, synthetic, and real-world stimuli are routinely used for his purposes. Tools used for this purpose include Siemens EDA (ex-Mentor Graphics) Questa [53], Synopsys VCS [54], and Cadence Incisive [55].

Depending on target implementation technology, e.g., ASIC or FPGA, a place and route phase takes place following RTL synthesis. The former (place) allocates various SoC blocks (all the way down to discrete transistors) to specific coordinates in a 2D or 3D space, whereas the latter (route) selects the specific routes that various nets take to connect the SoC components (from blocks all the way down to transistors). Various electronic design automation (EDA) tools are used in this phase including Siemens EDA’s Calibre [56], Synopsys’ Silicon Compiler [57], and Cadence’s Innovus [58]. These tools optimize for user parameters, e.g., speed, area, and power, supplied in the form of constraints or intents. As the SoC design moves through lower levels of abstractions, various verification, validation, and equivalence checking processes are usually executed to ensure consistency across the phases and with the original functional model.

The final layout (in GDSII format) can then go to a Foundry for “tapeout”. Chips are subsequently tested, and then diced off the wafer and packaged. Final packaged SoCs are graded through various tests, e.g., to determine speed grades that account for fabrication variations or downgraded because of faults in parts of the SoC. The physical layout of a commercial SoC is provided as illustration in [59].

### 3.2. The Role of the Intellectual Property (IP) Industry

**Figure 3:**
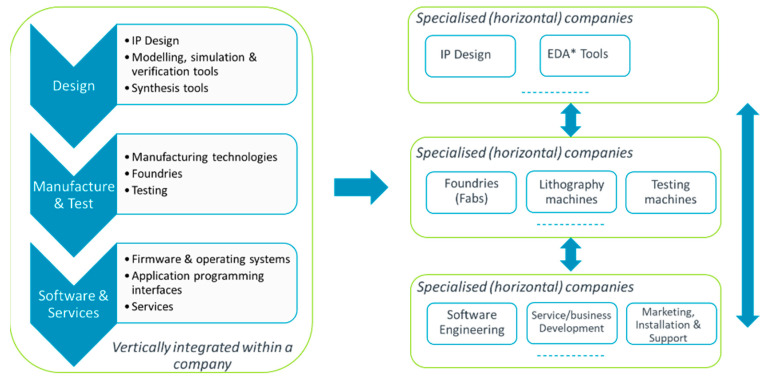
Specialization of SoC development tasks. (* points to the place of EDA Tools in diagram).

The relentless increase in the complexity of modern SoCs has led over the years to an increased level of specialization at various supply chain stages, as shown in Figure 3.

The intellectual property (IP) market, in particular, allowed for the development of high-quality, high-performance IP blocks, e.g., processor cores, interconnect, optimized memory blocks, standard, and customized library cells, to suit the needs of various parties in the supply chain. The latter would license such pre-designed pre-verified IP blocks and configure them to suit their own needs. This extreme specialization allowed the semiconductor industry to continue to deliver higher and higher customer value at the same or even lower price, although the cost of SoC development has increased massively.

Complex software stacks are used and reused thanks to standard processor IPs, mostly following the Arm architecture these days. As stated above, software compatibility is often a deciding factor in the choice of a particular IP in a SoC design.

### 3.3. Brief Comparative Analysis of SoC Design Methodologies and Tools

The evolution outlined in earlier subsections indicates that whereas both graphic-based methods and text-like methods are being effective in providing designers with useful tools and techniques for developing novel electronic systems design, both of these categories need to handle a high level of abstraction in order to cope with increasingly complex functionality. They also need the ability to communicate with other tools, from the electronics domain and other areas, in order to address the increasing interdisciplinarity nature of products and services required by industry and the market. If we are to look at each category, the schematic-based tools cover the entire system design cycle, but Mathworks family tend to handle higher levels of abstraction (mathematical modelling), whereas the National Instruments family can cope better with the details of physical implementation, being particularly useful in mechatronics and robotics systems for example, where various sensors and actuators are involved. The text-based methods can perhaps more flexibly flow from abstract modelling to details of implementation, with a range of vendor specific libraries and tools being available to support the flow. All tools, however, need to accommodate increased system complexity, a variety of target devices for implementation and effective inter-tool communication, in order to be viable and to address the current challenges highlighted later in this paper.

SoCs are employed with various roles, generating (for example) signals for controlling power converters; such control does not require the computing power of a custom SoC as such but uses FPGAs for digital controller implementation. Tools for such digital systems design include HDL to design FPGA controllers for power converters and electrical drives. Some of the design code can be auto generated from Simulink models but most of it is manually written. The RTL code is simulated using tools such as Questa from Siemens EDA and Active-HDL from Aldec [60]. Aldec CTS tools are used for board level verification of the FPGA designs. This is a tool which compares the RTL simulation results with the behavior of the configured FPGA on a PCB. These tools prove the equivalence of the RTL code with the fully configured silicon, which is a means to prove the correctness of the synthesis process, the place-and-route process, and the generation of the configuration file. This demonstrates that SoC design require the use of various tools, from a range of categories, which can be used independently or in combination, ultimately increasing the confidence in correct operation and enhancing security of service of the end-product.

Using the model-based design (MBD) allows a top-down approach to SoC design. The basic functionality can be developed entirely offline before the partitioning of any of the functionality is decided or is even clear. The tools have been available recently to take a model of the whole system and generate the requisite code, either C/C++ for CPUs or VHDL/Verilog for FPGA fabric or ASIC. The advantage of MBD is that the model of the system components uses the same graphical language, providing the ability to target the execution of blocks of functionality needed to either of the two, a flexibility that reduces the turnaround time for implementing changes.

The flexibility of distributing the functionality as supported through targeted code generation extends to validation. The current tools allow for validation at various stages of development using model-in-the-loop (MIL), software-in-the-loop (SIL), processor-in-the-loop (PIL), and hardware-in-the-loop (HIL) simulations. These allow the gradual validation of the implementation, from the concept stage to the final product, using broadly the same tools, and integrating with third party tools from dedicated software houses or semiconductor manufacturers.

Programmable logic devices, such as FPGAs and FPGA SoCs (FPGAs with microprocessors on the same chip), have significantly impacted the history/development of low power electronics equipment. They allow a fast development from concept to experiment (fast prototyping) and low non-recurring engineering costs. Some current developments are taking significant advantage of such device characteristics, by pushing the high computational efforts on parallel acquisition channels to their limits. FPGAs and SoCs are a perfect fit for equipment such as the European X-ray free-electron laser, as reported in [61]. Furthermore, special applications can exploit the flexibility of HDL design styles (such as asynchronous structures), effectively transforming FPGAs into ASIC-like devices, where an architecture optimization tailored to the application is achieved, while maintaining low non-recurring cost. Time-to-space conversion is a good example of imaging application being implemented in FPGA rather than ASIC [62]. Time-to-digital converters (TDCs) constitute a relevant example of picosecond precision being achieved in time-measurement applications, as reported in [63].

Table 1 aims to summarize the brief comparison of SoC design methods presented in this subsection. This can be read in conjunction with a very informative table provided in [30] with respect to target technologies for digital system-on-chip implementation.

In summary, the EDA tools and platforms enable high-level design methods to be deployed as effective solutions for achieving faster, compact, and energy-efficient hardware design in the form of system-on-chip solutions, following a typical industrial electronics design flow and enabled by the IP industry. Such EDA environments enable software engineers to design system-level/chip-level solutions, and ultimately to design optimized and effective hardware implementations. This approach also enables the effective holistic modelling and functional simulation of complex systems based on SoC electronic controllers, with a plethora of applications such as: mechatronics [64,65], robotics [66,67], automation [68], security [69], power electronics [70], energy systems [71], electric drives [72], and IoT systems [73,74,75,76,77].

## 4. SoC Design Applications: Aerospace and Automotive as Case Study Areas

This section covers, as examples, two application domains where SoC methodologies and tools have been and continue to be an essential engine for technological progress: aerospace and automotive.

### 4.1. Aerospace Applications Domain

System-on-chip (SoC) technology has several applications in the aerospace industry which range from avionics systems to engine control systems and satellites.

Avionics systems are responsible for monitoring and coordinating the major aircraft functions, including navigation, communication, flight control, and engine control. Traditionally, these functions have been implemented using many separate electronic components, such as microprocessors, memory, analog-to-digital converters, and communication interfaces. It is now possible to integrate several such components into a smaller number SoCs, reducing the overall size and power consumption, while improving the reliability of civil and military aircraft avionics system. A single SoC can be used to implement the full range of avionics functionality in the case of unmanned aerial vehicles (UAVs), where size and weight are even more important than in the case of a manned aircraft.

SoCs can also be used to design a full authority digital engine control (FADEC) system for aircraft engines [78]. The main functions of a FADEC are to control the initial engine start and to optimize the engine operation throughout the flight. The engine thrust is regulated by adjusting parameters such as fuel flow, stator vane position, bleed valve position, etc. Other functions include general engine monitoring and safety features such as engine overspeed protection, stall prevention, and automatic shutdown in case of an emergency. Some FADECs include complex prognostics and/or health management (PHM) algorithms, which in some cases may employ artificial intelligence techniques.

To perform their functions, FADECs interface with multiple sensors for air pressure, engine speed, temperature, etc., distributed around the engine. A combination of microprocessors, A/D and D/A converters, and communication interfaces is necessary to implement the functionality outlined above. However, FADECs are generally mounted on the engine and are expected to achieve very high reliability while operating in harsh environments involving high altitude, high temperatures, vibration, lightning strikes, etc. As a result, the reliability of SoC technology, particularly in its application-optimized ASIC form, is an attractive implementation technology for this type of specialized equipment.

SoCs are commonly used by satellites for several functions including data collection from sensors and cameras, data compression, communication with ground equipment, altitude control (using sensors to detect the orientation of the satellite and adjusting its thrusters to maintain the desired orientation), and navigation (using sensors such as gyroscopes and accelerometers to calculate the satellite position and speed in orbit around the Earth). SoC technology can also be used for power management, allowing the satellite to optimize its power production by controlling the orientation of its solar panels and balancing this against its power usage. A SoC-based system can perform the necessary computations to decide which onboard equipment can be allowed to operate depending on the amount of power available from the solar panels and battery systems.

A common characteristic of system-on-chip architectures used in aerospace applications is redundancy. For instance, FADECs implement dual redundant architectures with two control units (a primary lane and a secondary lane). The secondary lane is a hot-standby unit which is always available to assume full engine control in the event of failure of the primary lane. This ensures the uninterrupted operation of the engine until the aircraft lands at its destination. Some satellites as well as most deep space interplanetary probes use triple modular redundancy (TMR) hardware architectures to help recover from “soft errors” (corruption of digital data without permanent failure of the digital components) which are caused by the effects of cosmic radiation on electronic equipment. In all aerospace applications, the architectural redundancy of the hardware is complemented by advanced error correction codes which use information redundancy to retrieve the original data from corrupted messages received via noisy communication channels. Some of the error correction codes are implemented in software while others are implemented directly by special hardware blocks in the SoC, depending on the cost target and the safety criticality of the application.

Generally, airworthiness certification is governed by three main documents. Complex digital hardware (which includes FPGAs, ASICs, SoCs, and digital control circuits composed of multiple components) is governed by a standard known as DO-254. Software development is governed by DO-178. These are the application programs running on the complex digital hardware mentioned above. Finally, certification of systems which include digital controllers as well as electromechanical hardware or hydraulic hardware or pneumatic hardware, or any combination of those is governed by ARP4754. These are discussed in [79].

There are additional standards which stipulate specific certification requirements for things like model-based design (DO-331), qualification of design tools used for software and hardware design (DO-330), etc. For instance, if Matlab/Simulink is used to create models which are then automatically translated to design code and the result is implemented in a custom SoC, then Simulink needs to be qualified in accordance with DO-330, the models need to be validated in accordance with DO-331, the resulting software needs to be verified in accordance with DO-178, while the SoC hardware needs to be designed and tested in accordance with DO-254. Finally, the overall system will undergo environmental testing (testing at relevant temperature, humidity, vibration, and electromagnetic interference) in accordance with DO-160.

In terms or aerospace safety, general safety aspects are governed by ARP4761. This stipulates a number of system safety assessments which have an impact on how thorough the validation and verification activities need to be at component level (including the SoCs). Generally, aircraft systems are classified on a scale with several levels which indicate the aircraft level effect of a failure in that system. This is the so-called design assurance level (DAL level) of the system and includes the following:

DAL A: Catastrophic effects—the failure will cause the loss of the aircraft and loss of life.

DAL B: Hazardous—the failure will cause injuries or fatalities without necessarily losing the aircraft.

DAL C: Major—the failure will cause discomfort and minor injuries.

DAL D: Minor—the failure will reduce the safety margins of the aircraft.

DAL E: No safety effect—no impact on aircraft operation.

The aim of the design process and the certification procedures is to ensure that failures of a DAL A system happen less often than 10^−9^ failures per hour of operation. Similarly, for DAL B systems, the target is <10^−7^ failures per hour of operation. The exact failure rate on a particular application may be set even lower (such as 10^−10^ or 10^−11^), depending on the type of aircraft, certification authority, etc.

The system DAL classification is flowed down to its subsystems and components. Components can have a different DAL classification compared to the higher-level system. For instance, a dual-redundant system will fail if both its “lanes of operation” fail. So, dual redundant DAL A system could be composed of two dissimilar DAL B subsystems. However, if the subsystems are identical, then it is possible to have a double failure due to a common mode failure. For instance, the same type of component is used which fails simultaneously in both subsystems at the same temperature. In such case, a DAL A system may be composed of two DAL A subsystems. There are also opposite examples where a DAL B system is composed of DAL A subsystems when the failure of either subsystem will cause the large system to fail.

The selection of tools for the design at component level (FPGA, ASIC, software running on them) is dependent on the DAL level of the component and is governed by the standards mentioned above (DO-254, DO-178, DO-330). Tool qualification is complex and expensive, which means that often design teams use the alternative allowed by DO-254 and DO-330, which is to involve independent tools to perform the verification of the design. For instance, in FPGA design the same VHDL code can be simulated by independent tools from independent providers, such as Questa from Siemens EDA and Active-HDL from Aldec [60]. This ensures that false simulation results due to simulator bugs can be spotted by comparison of simulation results of the two tools. Furthermore, hardware tests will be conducted in the real hardware to check (some of) the results from simulations.

SoCs are employed with various roles such as the navigation system, the communication system, the flight control system. These then generate signals for controlling power converters. Such systems typically use FPGAs for digital controller implementation. Typical hardware target are flash-based FPGAs (such as Smart Fusion, Igloo2 and PolarFire) and SoCs from Microsemi (part of Microchip now). This avoids the need for configuration memory scrubbing, which would be necessary with radiation-sensitive RAM-based FPGAs. RAM-based Xilinx or Intel (e.g., Altera) FPGAs and SoCs are not as widely used because they do not provide the level of radiation tolerance required in aerospace applications. Another aspect to mention is that such applications are not sensitive to FPGA power consumptions because they are not battery-operated mobile devices. In these parts of aerospace applications/equipment, FPGAs are operated at low to medium clock frequencies (40–50 MHz) because these do not involve high-bandwidth communication channel like telecommunication equipment. Another notable aspect is that normally the target devices are selected from the military temperature range (−55 °C, +125 °C), given that aerospace systems must operate at the low temperatures typical for high altitude.

A specific aspect of aerospace industry, when compared with automotive industry, is the relatively low manufacturing volume. A manufacturer like Airbus, Boeing, Bombardier, and Embraer will not manufacture more than a few tens of aircraft of a particular type per month. By comparison, a company like Ford will produce several thousand cars of a particular type per day. The large manufacturing volumes mean that investing in ASIC design is more likely to be economically viable for an automotive company than it is for an aerospace company. Therefore, more of the aerospace applications will use FPGAs and more of the automotive applications will use ASICs. However, some of the aerospace applications have special environmental requirements related to temperature and radiation tolerance, etc. In these cases, special ASICs may be used to ensure the expected levels of performance which go above the capability of commercial components. Sometimes, the bare ASIC die may be packaged together with RAM memory, EPROM memory and various other components. The result is multi-chip-module (MCM), which is basically a SoC, aiming to improve the design of FADECs (those used by Rolls-Royce engines, for example).

**Figure 4:**
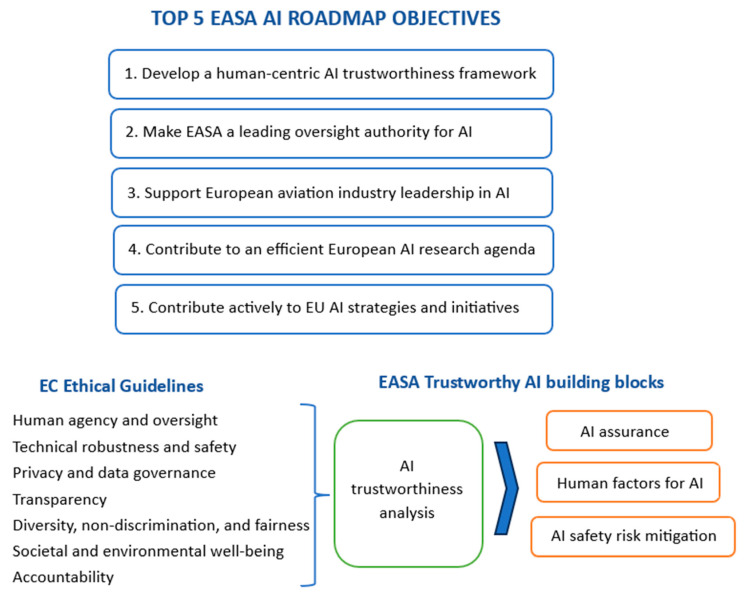
EASA AI Roadmap 2.0 [80].

Finally (for this section), an important aspect to cover is the use and regulation of AI in aerospace. Concerns and challenges related to the certification aerospace applications which would include elements of AI are summarized in [79]. A few interesting aspects are covered by the diagrams provided in this publication, if the reader is interested in more details. However, more general and more recent considerations about AI in aviation/aerospace are covered in a whitepaper from European Union Aviation Safety Agency (EASA)—a certification authority [80]. AI is an emerging technology that will affect multiple domains of the aerospace/aviation sector, to address its challenges such as air traffic volume increase or environmental standards. Some use case studies are provided, together with an overview of the rulemaking strategy anticipated by EASA, including specific aspects such as: (i) aircraft design, operation, production, maintenance; (ii) air traffic management, drones, aerodromes; or (iii) safety risk management. Figure 4 [80] presents the human-centric approach to application of AI in aviation.

The use of ML/AI in the aerospace industry is explored, including plans for flight critical applications such as navigation solutions. However, there are still some concerns in this respect, which include data-driven development, statistical/probabilistic outputs, repeatability, etc. Approaches to build confidence in the use of machine learning in flight critical applications are reported in [79], with solutions including combined evaluation of performance assurance and mitigations.

### 4.2. Automotive Applications Domain

Electronics and software in vehicles have seen an exponential rise in the last couple of decades, a trend set to continue. A high-end car sold 30 years ago would have had a handful of electronic control units (ECUs)-embedded computers effectively—whereas a similar one sold today would have in excess of 100 [81]. This may go a long way to explaining why SoC technologies are a relatively new phenomenon in the automotive industry: historically, functionality was implemented in specialized controllers next to the physical systems they were controlling, which from a PPAC perspective provided enough performance, good power control, efficient space use and low cost, with the cost of non-volatile memory being a major factor [82].

There have been three main drivers in the adoption of new electronics and software technology in the automotive industry, and therefore of SoCs: increased functional complexity, safety requirements, and development tools support.

In terms of functional complexity, the last decade saw two very significant areas of vehicle development: advanced driving assistance systems (ADASs), with their natural progression into autonomous driving (AD), and electric propulsion. While the latter saw an increase in the use of power electronics in vehicles, the former is extensively data-driven [83].

The Society of Automotive Engineers (SAE) defines in its standard “J3016 SAE Levels of Driving Automation” 6 levels, from Level 0 (lowest) to Level 5 (highest), with the Levels 0–2 being known in the industry as ADAS, and Levels 3–5 as AD. ADAS entails technologies that assist the driver in its actions, but the driver still has control and is responsible for driving. AD entails features that take over the responsibility of driving, with very limited driver interaction, if at all, and builds on the ADAS technologies that are necessary to achieve AD [84].

ADAS uses a wide range of sensors to assess the environment around the vehicle: radar, lidar, video, ultrasonic, GPS, etc. These sensors produce a vast amount of data that needs to be processed, sometimes exceeding 50 Gbps. This led to the concept of sensor fusion, where SoCs show their versatility. By using SoC technologies, different types of data streams can be processed efficiently in one place, reducing the need for expensive inter-ECU connectivity, and achieving a ‘fusion’ of the data such that relevant information is extracted quickly from the data and redundant information—data about the same feature of the environment from multiple sensors—is safely discarded. SoCs are perfectly suited for such applications, and such chips would contain communication transceivers for retrieving the data from the sensors, FPGA fabric for fast pre-processing of the raw data, parallel computing RISC paths for complex data processing, and processor cores for control and decision algorithms. For this purpose, SoCs typically come from semiconductor companies with historical automotive presence [85].

AD has a different focus in that it uses the data processed by the ADAS systems and utilizes a significant amount of ML/AI algorithms. Such processing requires a different mix of features to the ADAS SoCs, and unlike other ML/AI systems, it cannot rely on cloud computing power, it must be able to process the information efficiently and very fast. For this reason, new types of SoC dedicated to AD have been released recently, but by semiconductor companies historically associated with general computing, rather than with legacy automotive control [86].

Although not increasing exponentially in complexity, the area of telematics has acquired new features that proved fertile ground for SoC technologies. Apart from providing driving information using the global positioning system (GPS), telematics has added features related to the connected vehicle, whereby the vehicle exchanges information with the rest of the world through the Internet, predominantly via mobile connections, but also via short-range networks, such as to search for and guide to electric vehicle (EV) charging points, or to notify the fleet management of the vehicle status. This provides new challenges in communication security and has an impact on functional safety [87].

Another area of development in the automotive industry with relation to SoC and custom chips is the power management. How much power ECUs use during their ON state was a marginal concern in the past, although that has changed more recently with the EVs’ internal energy budget being very tightly controlled for range reasons. ECUs’ power consumption during their OFF state, however, was always an important consideration. New technologies have allowed the design and the proliferation of system basis chips (SBCs), which contain a number of analogue, digital, communication, and power electronics components on a single chip, allowing the control of power management across an ECU, as well as taking over some of the functionality typically implemented in the main microcontroller. Such components would be found in different chips in the past, but the SoC technologies enabled their consolidation in one chip, with benefits of cost saving, IP protection, and safety certification.

Some of the components used in EVs are radically different to the ones used in traditional non-EV vehicles. The high voltage (HV) batteries require specialized monitoring and control, achieved through dedicated cell supervisory circuit (CSC) chips. Such CSC chips contain ADC circuitry for voltage and current monitoring of the individual cells, power transistors for performing the balancing of the cell current, SPI circuitry with galvanic isolation for communication with the battery controller, as well as preprogrammed—but configurable—logic and diagnostics [88]. Utilizing such SoCs provide significant cost savings and standardized monitoring strategies. They are also safety-certified, streamlining the certification process for the whole system.

Another essential component in EVs is the electric motor control. The strategies needed for the control of electric motors require for their effective implementation dedicated FPGAs or SoCs with FPGA fabric: the most recent control algorithms can only be implemented on such devices. In the early years of EV development, the majority of such drivers were implemented using FPGAs. Competition with the other industries in the wake of the COVID-19 outbreak for FPGAs, as well as an increased maturity of the control strategies that became state-of-the-art, led to the design of specialized SoCs used for this purpose, at least at the level of the current loop. The speed loop is still generally executed on the main microcontroller.

Alongside the need for accurate motor control for propulsion, devices in modern vehicles may require special control strategies, which are addressed by SoCs that are dedicated to a specific application, such as valves and pump control for suspension systems or brushless DC motors in power-assisted steering, essentially for accurate inductive loads control.

With the release of ISO 26262 “Road Vehicles—Functional Safety”, which imposes various safety level requirements for all parts of a vehicle, the functional safety validation of a SoC that has been certified to the requisite safety level by the manufacturer is much more effective and cost-effective than the functional safety validation of a system composed of various components, not least due to IP concerns [89,90].

The adoption of SoC technologies in the automotive industry is also strongly linked with the availability of development tools for these chips. As a SoC is heterogenous in its composition, with various technologies that historically utilize completely different development processes under the same roof, its adoption in the automotive industry is conditioned by the existence of tools that integrate well with the prevailing processes.

The automotive industry uses the model-based design (MBD) paradigm extensively [91]. A data-flow description with control-flow additions helps to manage the complexity of a system by allowing the modelling of both the control strategy and the physical systems and environment it interacts with in the same development environment. It is therefore essential that any new technology, SoC included, needs to have the development tools to support this paradigm. Using the MBD allows a top-down approach to designing automotive systems, enabling the gradual validation of the implementation, CPU or HDL, from the concept stage to the real ECU, using broadly the same tools, and integrating with third party tools from dedicated software houses or semiconductor manufacturers.

**Figure 5:**
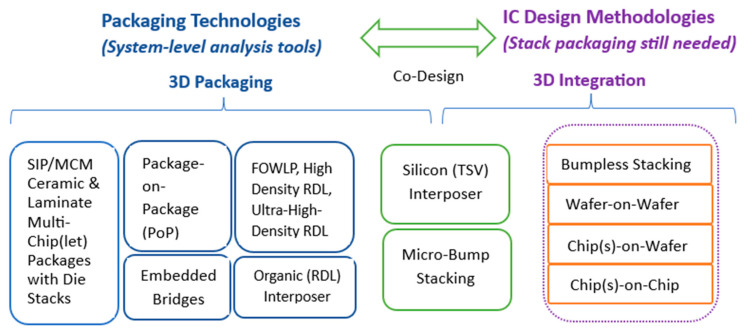
3D technologies—IC design and packaging [93].

Due to such progress, the development tools and validation environments provided or supported by SoC designers and manufacturers have reached a level of maturity that enabled the automotive industry to gradually move towards the extensive utilization of SoCs [92]. However, an outstanding issue rests with packaging, which has specific challenges for automotive. There are several technologies available, their selection and use being significantly influenced by thermal dissipation, reliability, and cost. The chips in the vehicle are expected to behave reliably, with high immunity to vibration and dissipating heat appropriately (not requiring a lot of cooling), while also being relatively lightweight and small in dimensions. Therefore, 3D technologies and stacking die packaging technologies (Figure 5, Cadence, [93]) are not easily adopted in vehicles; however, they could be used in automotive monitoring centers. Also, the denser the content (circuits) on chips, the higher the risk of cosmic effects affecting functionality [93].

Finally, recent developments of SoC applications to the automotive domain relate to the inclusion of various sensors in the SoCs (such as for self-driving cars), which have to be reliable, affordable and sufficiently miniaturized, thus introducing the concept of light detection and ranging (LIDAR) on a chip [94].

## 5. Challenges and Future Trends in SoC Design

This section discusses SoC design methods by looking at some of the current challenges and identifying interesting new developments and future trends, on the roadmap from the early stages of Moore’s Law [1], through the IC design major transformations enabled by the significant contributions brought to EDA tools [95], and process technologies leading to the current state-of-the-art of high-numerical-aperture extreme-ultraviolet (EUV) lithography as the current state-of-the-art of the transistor miniaturization approach [96].

### 5.1. Current Challenges Alongside Technology Enablers

The need for computing is increasing fast in all areas of life, most applications becoming interdisciplinary nowadays, whether looking at robotics, mechatronics, healthcare applications, smart cities, intelligent transportation, energy systems and smart grids, sustainable development, or mobile devices and applications—and the list can continue. Therefore, further development work will have to focus towards ensuring that electronic system design methods and tools are addressing the current challenges of the related industries, such as the growing need for accommodating multi-domain integration, lower power consumption, and higher performance versus reduced size/compact implementation, increased level of smartness, big data capacity, privacy and security, e.g., cloud versus local processing balance.

If we were to summarize the current challenges for digital electronic systems into one word, that will have to be ‘smart’. There is a clear need for smart electronic systems to be embedded in modern complex engineering systems, with a strong emphasis being placed on the importance of coping with the increasing number of smart monitoring and control tasks (diagnosis, prognosis, fault tolerant capabilities, power flow optimization, handling large volume of data, or implementing various AI control algorithms, to name just a few) [30].

In terms of implementation targets, despite of a certain number of limitations like cost, limited analog interfaces, and a relatively long designer learning curve for optimal use compared to pure software engineering, one of the most suitable technologies to implement such smart controllers is FPGA as SoC for rapid prototyping, leading to ASIC implementation, where economically viable. FPGA devices are not only able to manage complex algorithm processing, optimization or HIL/Digital Twin, but they can also help to accelerate their execution by parallelizing into customized hardware accelerators several computationally demanding subtasks.

Based on their highly performing FPGA fabric, the architecture of such devices can relieve the processing system from low-level time-consuming tasks. As FPGAs feature a large number of I/Os, they provide the added advantage of enabling easy communication with a complex system to be controlled. Furthermore, FPGAs can now embed feature-rich operating systems, thus rendering an FPGA-based smart controller into an effective software-defined edge computing platform, with the ability to address new challenges inferred by data driven approaches in terms of complexity, storage, and processing [30].

Another enabler of the recent advancements in SoC design is constituted by open-source EDA tools available that can be used to design industrial quality digital circuits. The ongoing challenge is the lack of full end-to-end open-source tools, but recent developments demonstrated some success, such as the use of OpenLane framework (based on the OpenROAD tool 2), where a SoC design was demonstrated [97,98]. An interesting approach is presented in [99], which demonstrates that the combined use of open-source EDA tools and ML techniques can lead to better design optimization, which in this case was achieved earlier in the design flow, at RTL level, based on the model-driven ML techniques.

### 5.2. Emerging Needs: 4.0 Industrial Revolution and Cross-Disciplinary Applications

**Figure 6:**
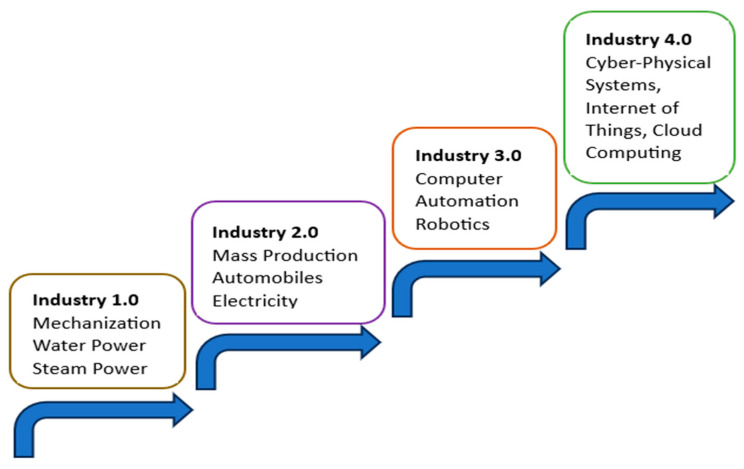
Industrial revolutions roadmap to Industry 4.0 [100].

The fourth industrial revolution, presented as the current step in the historical progress and development of industrial revolutions in Figure 6 [100], is marked by smart systems/factories, autonomous industrial cyber-physical systems, IoT, and cloud computing.

The authors of [90] highlight that a smart system is not just a collection of intelligent components, but a system of smart components designed to interact and cooperate. Therefore, the electronic systems design methods of the future need to address the synergy of big data, computing power, 5/6 G, robotics, machine learning, digital twins (including a wider range of application domains, such as construction [101,102]), in a context where the IoT enriched with AI becomes the state-of-the-art technology to support a user-centric ecosystem. Therefore, design engineers and companies need to be ready to migrate their design methods, product offering and production infrastructure to Industry 4.0 compliant solutions for smart cities, IoT, or smart microgrids, to name just a few.

**Figure 7:**
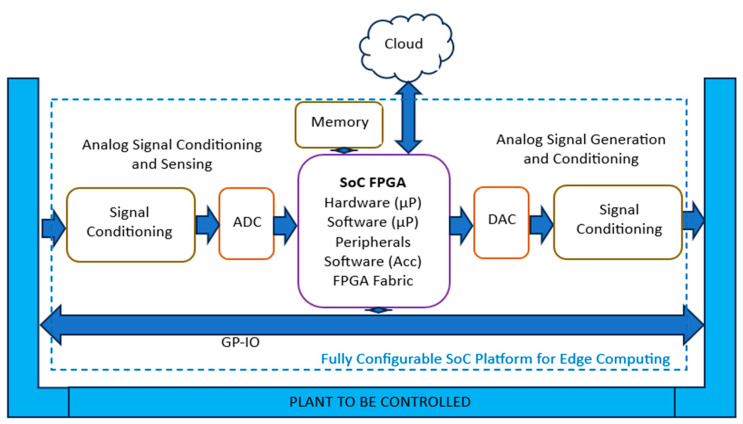
SoC platform for edge computing [104].

Some emerging examples of smart systems embarked on the current industrial revolution are well-captured in [103]. This article reinforces the message of the ‘right recipe for smart’ consisting of IoT enriched with AI. Real life examples highlighted in the paper include smart cities (e.g., Pittsburgh in the USA tackling snow, based on edge computing and AI-enriched IoT applications based on technology developed by Metro21), IoT technology making use of a network of nanosatellites (e.g., by French company SigFox), or contactless deliveries developed in response to the COVID-19 pandemic [104]. Considering such complex applications of the electronic systems of the future, with challenges to satisfy power efficiency, flexibility, scalability, communication, and integration, the system-on-chip emerges as an ideal solution, enabling the designer to take advantage of the inherent parallelism offered by many algorithms and allowing the exploitation of hardware accelerators and dedicated peripherals in conjunction with CPU cores enabling ML/AI algorithms and connectivity, as shown in Figure 7 [104].

### 5.3. Future Trends on Chip Design—Holistic Systems Thinking and AI/ML-Enabled Design

An interesting publication [105] refers to recent challenges of climate change, coupled with biodiversity crisis and the recent COVID-19 pandemic, all with world-wide effect, reminding us of the fragility of our existence as humans. In the same article, the author highlights the consequent complex interdependencies between natural systems and engineering systems, with significant impact on public health and wellbeing. In this context, an argument is being raised that a change in thinking is required, when a cross-disciplinary holistic approach and system thinking/design approach are more relevant than ever before.

Natural systems behavior is mirrored more and more in electronic systems design through the use of AI and ML in a rapidly increasing range of applications. However, as highlighted in [106], a step change is needed in terms of hardware implementation in order to address the computational needs of AI-based systems, as traditional CPUs are not sufficiently powerful platforms for many AI applications. Increased efficiency is expected to come from a new breed of microprocessors tailored to the needs of AI in the coming decade. Physical limitations of chip physics (to 1 nm, about 10 atoms) [107], when Moore’s Law of transistor scaling [1] cannot be applied any more, are pushing for new materials and architectural innovations. For instance, chip architectures are rethought by many companies to be inspired closer by the interconnected structure of the biological brain, leading to the concept of a chip built for AI applications that could be called an AI chip. Neuromorphic SoCs are examples of this trend in the design of electronic system-on-chips [106].

**Figure 8:**
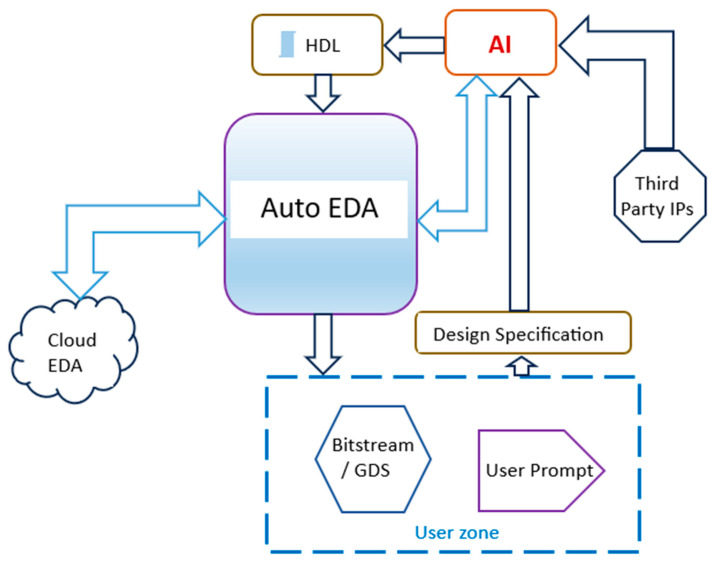
The role of AI in SoC design [108].

So, AI started to play a growing role in the design of SoCs, as generative AI is harnessed for optimized architectural space exploration, optimized place and route, code optimization, autocompletion of code, and at least partial automatic code generation [107,108]. This role can be visualized as diagrammatic representation in Figure 8 [108].

At the same time, ML/AI techniques are already used to develop methods for the combinational circuit generation [109]. Recently, advanced machine learning techniques have also been proposed for accurate power modelling, enabling optimization of power distribution at chip level and the reduction in power consumption [110]. The other use of ML/AI can be to verify the level of optimization of an intended chip design, by checking the quality of the HDL code even before the synthesis stage [111]. In terms of supporting ML/AI and making it work in conjunction with EDA tools, graphics processing units (GPUs) are playing a pivotal role as enablers in accelerating ML/AI applications these days, providing increased computing power [112].

### 5.4. Generative Design; Prompt Engineering

Generative design is a new concept in engineering design [113], which represents the latest trend in EDA tools evolution. It is based on the principle of AI optimization, enabled by the software packages used for engineering system design. However, instead of using the engineer to build up various designs in order to achieve product optimization, these advanced EDA tools will generate various versions of the design, achieving different levels of optimization. The EDA tools are therefore evolving into even more complex aids to the design engineer, still carrying some limitations though due to their performance being based on the environment set up by the engineer and the constraints imposed as learning point for the tools. The engineer still has a role, but this has evolved into that of understanding and selecting the best optimized alternative from the computer-generated designs, with the major advantage that there is no need for an in-depth knowledge of the specific details of the complexities of the designed product. Initially, this kind of approach/technique is applicable to specific single product designs, but more complex products and systems are expected to be designed in this way in the future [114,115].

Prompt engineering is defined as a comprehensive discipline within the wider field of AI, which involves a systematic design, refinement, and optimization of prompts to a generative AI engine, e.g., a large language model (LLM) and underlying data structures. The LLMs are AI models designed to understand and generate text like human language, based on large input data [116]. Their use guides AI systems towards achieving more specific outputs, facilitating effective interaction between humans and AI, which is expected to revolutionize engineering design. Prompts as part of prompt engineering are continuously evaluated and categorized to ensure their relevance and effectiveness. It is also important to maintain an up-to-date prompt library, fostering efficiency, accuracy, and knowledge sharing among AI professionals [117,118,119]. In a recent survey carried out by IEEE on key technology predictions for 2023 and beyond, 98% of respondents said that AI-powered autonomous collaborative software and mobile robots will play a key role in automating processes and tasks [120]; hence, SoC design methods and tools will need to be more AI-friendly. The use of AI in chip design has already started to cause heated debates and disagreements between experienced designers and AI users, for example in relation to reinforcement learning [121].

## 6. Summary

This paper reviewed SoC design tools and techniques for the integrated development of embedded digital electronic systems, including their main features, evolution, challenges, and trends for the future. Such methods make use of high-level languages and enable fast, compact, and power-efficient hardware implementation as system-on-chips. SoC development follows a design cycle starting from an idea that is then progressed through design and functional simulation stages, design space exploration, then through synthesis, timing analysis, and verification to complete system implementation. Through higher levels of abstraction and increased automation, the development cycle is completed in an increasingly efficient time cycle, with advantages such as high degree of flexibility and high confidence in the correct ‘first time’ operation of the system/product. Wide compatibility of the design with respect to multiple EDA tools and implementation target technology is assured. The IP industry has played a major role in the development of the SoC industry and will continue to do so, with a plethora of applications such as robotics, automation, security, power electronics, electric drives, aerospace, and automotive.

Current challenges of design methods and tools for SoCs, such as the growing need for accommodating multi-domain integration, lower power consumption, and higher performance versus reduced size/compact implementation, increased level of smartness, big data capacity, privacy, and security, were then briefly reviewed. Future trends and technology enablers, including smart systems, digital twins, hardware-in-the-loop (HIL) testing, open-source EDA tools, ML/AI including LLMs and prompt engineering, were introduced.

Overall, AI-assisted SoC design and implementation is increasingly commonplace and will play a bigger role going forward. Longer term, generative designs are likely to emerge on a wider scale as a new trend, with the role of the engineer shifting towards the analysis, interpretation, and optimized selection of designs generated by sophisticated EDA tools, with prompt engineering likely to bring large benefits of AI into the SoC design processes.

## Figures and Tables

**Table 1:** Comparative summary of SoC design methodologies.

Criteria\Method	Graphical	Text	Mixed	Custom
algorithm complexity	●○○	●○○	●●○	●●●
high accuracy	●○○	●●○	●●●	●●●
high speed/parallelism	○○○	●○○	●●○	●●●
short development time	●●●	●●○	●○○	○○○
high design flexibility	●○○	●●○	●●○	●●●
ease of learning	●●●	●●○	●○○	○○○
low development cost	●●●	●●○	●○○	○○○

Score: ○○○: poor, ●○○: medium, ●●○: good, ●●●: very good.

## Data Availability

Data are contained within the article.
